# Developing a practical machine learning model to predict post implantation syndrome after endovascular aneurysm repair

**DOI:** 10.1186/s42155-026-00668-w

**Published:** 2026-03-18

**Authors:** Jinhua Zhang, Dong Yang, Lei Zhang

**Affiliations:** 1 The First People’s Hospital of Foshan (The Affiliated Foshan Hospital of Southern University of Science and Technology), School of Medicine, Southern University of Science and Technology, Foshan, Guangdong China; 2 Guangzhou AID Cloud Technology Co., LTD, Guangzhou City, 510000 China

**Keywords:** Endovascular aneurysm repair, Post-implantation syndrome, Machine learning

## Abstract

**Background:**

Post-implantation syndrome (PIS) is recognized as a systemic inflammatory response following endovascular aneurysm repair (EVAR), characterized by a high frequency of occurrence and the capacity to provoke cardiovascular complications and extend the duration of hospitalization. The objective of our study is to construct a predictive algorithm through the application of machine learning (ML) techniques to forecast the onset of PIS subsequent to EVAR procedures.

**Methods:**

The data of 618 patients were retrospectively retrieved from the Electronic Health Record (EHR) system of Foshan First People’s Hospital, covering the period from January 2018 to December 2022. Least absolute shrinkage and selection operator (LASSO) regression is used for data preprocessing and variable selection. Eight ML models are developed to predictive PIS after EVAR. The area under the receiver operating curve (AUC), F1-score, accuracy, sensitivity, and specificity were evaluated as the model performances.

**Results:**

According to the exclusion criteria of 618 patients, 594 patients were finally included in the statistical analysis, and the incidence rate of PIS was 16.8%. Our research results show that there are 11 features that predict risk factors for PIS, including intraoperative use of etomidate, muscle relaxants, polyester endograf (knitted process), polyester endograf (woven process), glucocorticoids, phenylephrine, platelet count, age, absolute neutrophil count, surgical duration, and creatinine. The linear discriminant analysis (LDA) model performs the best among prediction models, with an AUC of 0.794, F1 score of 0.438, sensitivity of 0.7, specificity of 0.697, and accuracy of 0.697.

**Conclusion:**

Our study selected 11 preoperative and intraoperative variables to develop a ML model based on LDA for predicting PIS after EVAR and the model may help assist clinical decision-making.

**Supplementary Information:**

The online version contains supplementary material available at 10.1186/s42155-026-00668-w.

## Introduction

Abdominal aortic aneurysm (AAA) is a relatively common disease. It is reported that the incidence rate of men over 65 years old is as high as 8% [1]. When the diameter of AAA is greater than 5–5.5 cm, the risk of rupture exceeds the risk of repair, indicating the need for repair surgery [1, 2]. PIS is considered the systemic inflammatory reaction after the repair of intravascular aneurysms [3, 4], which is a very common postoperative complication. It is reported that its incidence rate is between 14 and 60% [5, 6]. Many studies have found that PIS increases cardiovascular events in patients [7], prolongs hospital stay [8], and is considered a moderate complication after surgery [9, 10]. Arnaoutoglou et al. found that PIS was associated with the occurrence of major cardiovascular adverse events. PIS was an independent risk factor for cardiovascular adverse events after EVAR, and the incidence rate in the PIS group was 4–5 times higher than that in the control group [3, 11]. There are also literature reports that PIS may be related to an increase in the thrombosis rate after thoracic aortic endovascular repair surgery [12]. Moreover, severe PIS may lead to pulmonary dysfunction, severe cardiovascular events, renal dysfunction, and even multiple system organ failure [13]. Therefore, analyzing the risk factors of PIS and constructing predictive models is of great significance for preventing postoperative complications of EVAR. However, there is currently a lack of effective risk classification and prediction models for PIS.

ML models are typically developed using extensive data sourced from EMR systems. Their profound capacity for handling big data enables these models to apprehend intricate nonlinear associations, including hitherto undiscovered correlations within extensive datasets, thereby facilitating a more comprehensive exploration of clinical data [14]. In the context of high-dimensional data, ML demonstrates superior predictive capabilities compared to traditional scoring systems when forecasting a range of diseases and clinical conditions [15–17]. In our study, we conducted a retrospective analysis of our hospital’s data from 2018 to 2022 using ML techniques. Our objective was to develop a ML model to predict PIS, with the aim of facilitating early clinical intervention to mitigate postoperative complications.

## Material and methods

### Study cohort and data collection

**Fig. 1:**
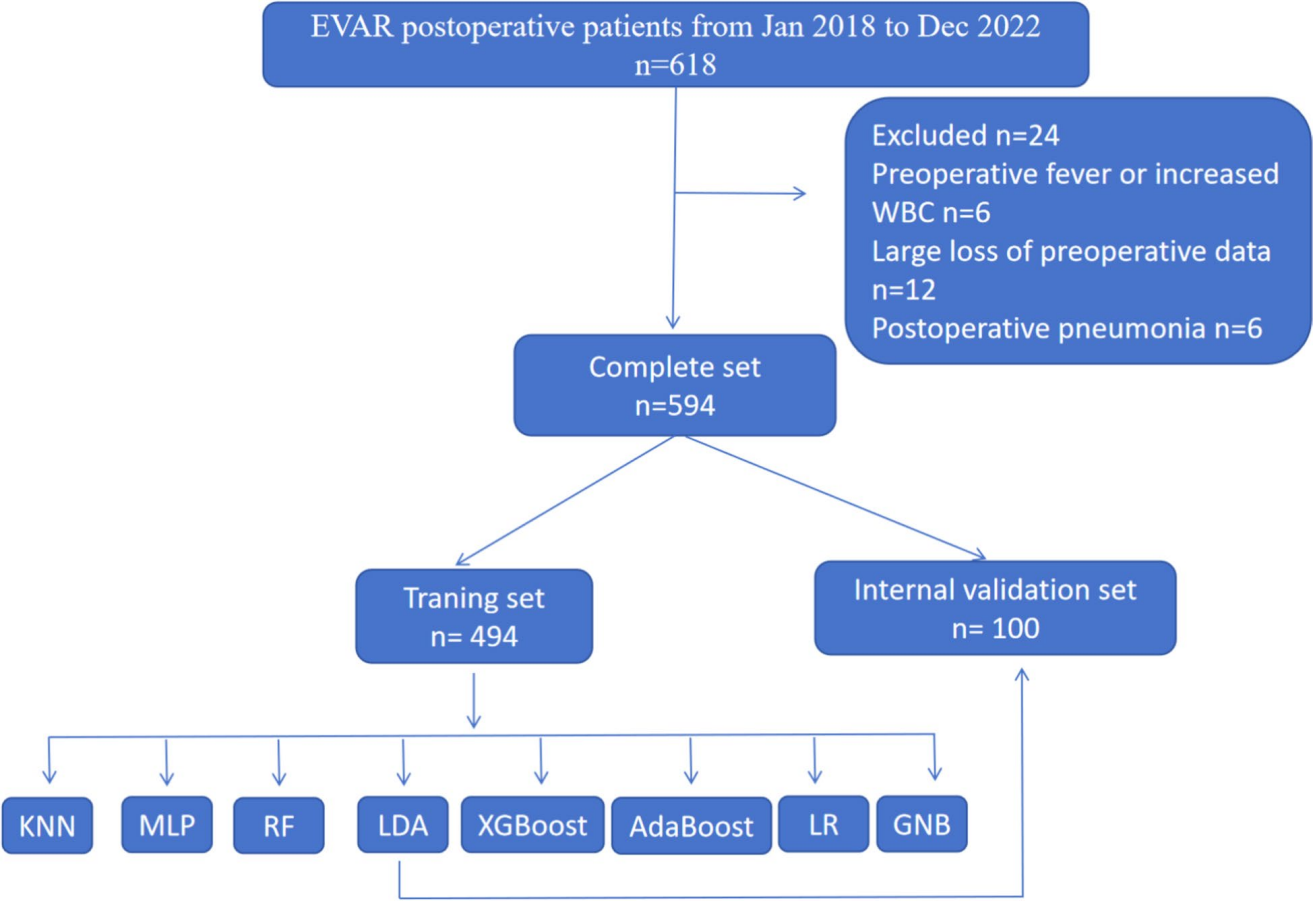
Flow chart of patient enrollment. WBC, white blood cell count; KNN, K Nearest Neighbors; MLP, Multilayer Perceptron; RF, RandomForest; LDA, Linear Discriminant Analysis; XGBoost, eXtreme Gradient Boosting; AdaBoost, Adaptive Boosting; LR, Logistic Regression; GNB, Gaussian Naive Bayes

This study was conducted and reported in accordance with the Transparent Reporting of a multivariable prediction model for Individual Prognosis Or Diagnosis (TRIPOD) statement. The study protocol was approved by the Ethics Committee of Foshan First People’s Hospital on 17 July 2023 (No. [2023]147) and registered at the Chinese Clinical Trial Center (ChiCTR2400081477). This study included patients who underwent EVAR treatment at Foshan First People’s Hospital from January 2018 to December 2022 as the training set, and from November 2023 to December 2025 as the time-series validation set. The exclusion criteria used were (1) clinical and/or laboratory evidence of preoperative infection, including increased white blood cell count (WBC > 10.000/µl) and fever; (2) age < 18 years old. A total of 618 patients were recruited into the study, and 594 were ultimately included in the statistical analysis, of which 80% were included in the training set and 20% were in the internal validation set. They were divided into PIS group and non-PIS group according to PIS diagnostic criteria, as shown in Fig. 1.

### Primary outcome

The primary outcome of our study was defined as PIS within 3 days after EVAR, regardless of any events that occur from 3 days later until discharge. PIS was defined in accordance with the definition of systemic inflammatory response syndrome (SIRS) by the presence of fever (continuous temperature > 38 °C) and leukocytosis (white blood cell (WBC) > 12,000/µl). Patients with other possible causes of fever and/or leukocytosis (such as urinary tract infection (UTI), pneumonia, sepsis, or positive blood culture) were excluded through medical record review, laboratory testing, and imaging examination [18].

### Variable selection

The study encompassed a total of 85 influencing factors, comprising 49 preoperative factors and 36 intraoperative factors. Characteristics were collected through the electronic medical records, including demographic characteristics, past and personal history, and preoperative laboratory data. Intraoperative data includes events, anesthesia methods, medications, fluids, blood transfusions, bleeding, urine volume, anesthesia duration, stent implantation status, etc. (Tables 1 and 2).

**Table 1:** Preoperative characteristics of training set patients

Characteristics	All population (*N* = 594)	no-PIS (*N* = 494)	PIS (*N* = 100)	*p-*value
Age (y)	69.0 [61.8; 74.5]	69.3 [62.7; 74.8]	66.8 [57.6; 73.2]	**0.022**
Weight (kg)	64.0 [57.0; 71.0]	64.0 [58.0; 71.0]	64.0 [55.0; 71.5]	0.460
Height (cm)	165 [161; 170]	166 [161; 170]	165 [160; 170]	0.152
BMI (kg/m^2^)	23.6 (3.53)	23.6 (3.41)	23.6 (4.10)	0.996
Gender				1.000
Male	524 (88.2%)	436 (88.3%)	88 (88.0%)	
Female	70 (11.8%)	58 (11.7%)	12 (12.0%)	
ASA classification				0.791
P1/P2	115 (19.5%)	97 (19.8%)	18 (18.0%)	
P3/P4/P5	476 (80.5%)	394 (80.2%)	82 (82.0%)	
NYHA classification				0.713
I	215 (36.9%)	179 (37.0%)	36 (36.4%)	
II	340 (58.3%)	280 (57.9%)	60 (60.6%)	
III/IV	28 (4.80%)	25 (5.17%)	3 (3.03%)	
Emergency or elective (*n*)				0.687
Emergency	56 (9.43%)	45 (9.11%)	11 (11.0%)	
Elective	538 (90.6%)	449 (90.9%)	89 (89.0%)	
Hypertension (*n*)				0.425
No	356 (59.9%)	292 (59.1%)	64 (64.0%)	
Yes	238 (40.1%)	202 (40.9%)	36 (36.0%)	
Diabetes (*n*)				1.000
No	549 (92.4%)	457 (92.5%)	92 (92.0%)	
Yes	45 (7.58%)	37 (7.49%)	8 (8.00%)	
Smoking history (*n*)				0.406
No	452 (82.8%)	375 (83.5%)	77 (79.4%)	
Yes	94 (17.2%)	74 (16.5%)	20 (20.6%)	
WBC (10^9^/L)	7.80 [6.33; 9.43]	7.71 [6.09; 9.40]	8.09 [6.66; 9.66]	0.050
RBC (10^12^/L)	4.25 [3.85; 4.68]	4.24 [3.85; 4.71]	4.28 [3.84; 4.67]	0.672
Lymphocyte ratio	0.20 [0.15; 0.26]	0.21 [0.15; 0.26]	0.19 [0.14; 0.25]	0.125
HGB (g/L)	127 [115; 140]	128 [114; 140]	127 [117; 140]	0.962
Neutrophil ratio	0.68 [0.61; 0.75]	0.68 [0.61; 0.74]	0.68 [0.62; 0.77]	0.275
Platelet (10^9^/L)	227 [189; 284]	224 [185; 278]	238 [200; 303]	**0.030**
Absolute monocyte count (10^9^/L)	0.56 [0.43; 0.73]	0.55 [0.43; 0.72]	0.61 [0.43; 0.80]	0.128
Hct (%)	0.39 [0.36; 0.43]	0.39 [0.36; 0.42]	0.39 [0.36; 0.43]	0.884
Absolute lymphocytes count (10^9^/L)	1.52 [1.12; 1.96]	1.52 [1.11; 1.96]	1.50 [1.15; 1.97]	0.875
Absolute basophil count (10^9^/L)	0.03 [0.02; 0.05]	0.03 [0.02; 0.05]	0.04 [0.03; 0.05]	0.128
Absolute eosinophil count (10^9^/L)	0.19 [0.10; 0.36]	0.19 [0.10; 0.35]	0.18 [0.08; 0.36]	0.314
Absolute neutrophil count (10^9^/L)	5.08 [3.80; 6.58]	5.00 [3.72; 6.53]	5.39 [4.29; 6.68]	**0.020**
APTT (s)	27.5 [25.4; 29.3]	27.4 [25.4; 29.2]	27.7 [25.6; 30.1]	0.230
Fbg (g/L)	3.36 [2.73; 4.29]	3.36 [2.73; 4.25]	3.29 [2.73; 4.34]	0.994
PT (s)	11.7 [11.1; 12.5]	11.7 [11.1; 12.4]	11.8 [11.2; 12.6]	0.191
TT (s)	16.6 [15.9; 17.5]	16.7 [15.9; 17.5]	16.4 [15.6; 17.2]	0.104
INR	1.00 [0.95; 1.06]	1.00 [0.95; 1.06]	1.01 [0.96; 1.09]	0.229
Calcium (mmol/L)	2.20 [2.11; 2.28]	2.20 [2.10; 2.29]	2.19 [2.12; 2.28]	0.837
Potassium (mmol/L)	4.04 [3.76; 4.28]	4.03 [3.75; 4.28]	4.06 [3.79; 4.30]	0.604
Sodium (mmol/L)	139 [137; 141]	139 [137; 141]	139 [136; 141]	0.877
Chloride (mmol/L)	102 [99.9; 105]	103 [99.9; 105]	102 [100; 104]	0.226
r-GT (U/L)	30.5 [23.0; 49.0]	31.0 [23.0; 49.0]	28.0 [22.0; 50.0]	0.444
AGR	1.50 [1.30; 1.60]	1.50 [1.30; 1.60]	1.50 [1.30; 1.70]	0.592
ALT (U/L)	16.0 [11.0; 23.0]	16.0 [11.0; 23.0]	15.0 [11.0; 24.0]	0.738
AST (U/L)	18.0 [15.0; 23.0]	18.0 [15.0; 23.0]	17.0 [14.0; 21.0]	0.147
ALP (U/L)	76.0 [64.0; 95.0]	76.0 [64.0; 95.0]	80.0 [65.0; 92.0]	0.848
AAR	1.10 [0.90; 1.40]	1.10 [0.90; 1.40]	1.10 [0.90; 1.40]	0.349
DBILI (µmol/L)	3.20 [2.40; 4.40]	3.30 [2.40; 4.60]	3.10 [2.40; 4.10]	0.229
IBIL (µmol/L)	6.35 [4.50; 8.90]	6.40 [4.40; 9.00]	6.30 [4.70; 8.40]	0.642
TBIL(µmol/L)	9.60 [6.90; 13.2]	9.70 [6.90; 13.4]	9.20 [7.30; 11.8]	0.453
Glu (mmol/L)	6.46 [5.38; 8.04]	6.39 [5.29; 7.99]	6.62 [5.72; 8.12]	0.296
Creatinine (µmol/L)	84.0 [68.8; 110]	85.0 [70.0; 109]	77.0 [65.0; 111]	0.082
Albumin (g/L)	40.4 [37.8; 42.8]	40.3 [37.6; 42.8]	41.0 [38.0; 42.8]	0.358
Globulin (g/L)	27.4 [24.7; 30.2]	27.4 [24.7; 30.4]	27.3 [24.0; 30.1]	0.913
Urea (mmol/L)	6.18 [4.98; 7.94]	6.17 [4.98; 8.07]	6.22 [5.01; 7.55]	0.788
Uric acid (mmol/L)	383 [300; 462]	385 [306; 470]	374 [286; 440]	0.243
Total protein (g/L)	68.0 (6.06)	68.0 (6.13)	68.1 (5.72)	0.927
Total cholesterol (mmol/L)	4.38 [3.73; 5.14]	4.38 [3.72; 5.20]	4.35 [3.88; 4.95]	0.828

Data were expressed as mean (SD), median (IQR), and frequency (proportion). Bold data indicates significance at *p* < 0.05

*WBC* white blood cell, *RBC* red blood cell, *HGB* hemoglobin concentration, *APTT* activated partial thromboplastin time, *PT* prothrombin time, *TT* thrombin time, *INR* international standardized ratio, *r-GT* r-glutamyl transferase, *AGR* albumin to globulin ratio, *ALT* alanine transaminase, *AST* aspartate amino transferase, *ALP* alkaline phosphatase, *AAR* aspartic acid to alanine ratio, *DBILI* direct bilirubin, *IBIL* indirect bilirubin, *TBIL* total bilirubin

**Table 2:** Comparison of intraoperative factors between two groups

Characteristics	All population (*N* = 594)	no-PIS (*N* = 494)	PIS (*N* = 100)	*p-*value
Anesthesia method (*n*)				0.811
Endotracheal intubation	479 (80.6%)	397 (80.4%)	82 (82.0%)	
Laryngeal mask	115 (19.4%)	97 (19.6%)	18 (18.0%)	
Glucocorticoids (*n*)				0.119
No	529 (89.1%)	435 (88.1%)	94 (94.0%)	
Yes	65 (10.9%)	59 (11.9%)	6 (6.00%)	
Dexamethasone (*n*)				1.000
No	582 (98.0%)	484 (98.0%)	98 (98.0%)	
Yes	12 (2.02%)	10 (2.02%)	2 (2.00%)	
Methylprednisolone (*n*)				0.223
No	550 (92.6%)	454 (91.9%)	96 (96.0%)	
Yes	44 (7.41%)	40 (8.10%)	4 (4.00%)	
Blood transfusion (*n*)				0.454
No	543 (91.4%)	454 (91.9%)	89 (89.0%)	
Yes	51 (8.59%)	40 (8.10%)	11 (11.0%)	
Phenylephrine (*n*)				0.132
No	448 (75.4%)	379 (76.7%)	69 (69.0%)	
Yes	146 (24.6%)	115 (23.3%)	31 (31.0%)	
Norepinephrine (*n*)				1.000
No	305 (51.3%)	254 (51.4%)	51 (51.0%)	
Yes	289 (48.7%)	240 (48.6%)	49 (49.0%)	
Hypotensor (*n*)				0.372
No	485 (81.6%)	407 (82.4%)	78 (78.0%)	
Yes	109 (18.4%)	87 (17.6%)	22 (22.0%)	
Neostigmine (*n*)				0.320
No	487 (82.0%)	409 (82.8%)	78 (78.0%)	
Yes	107 (18.0%)	85 (17.2%)	22 (22.0%)	
Dexmedetomidine (*n*)				1.000
No	437 (73.6%)	363 (73.5%)	74 (74.0%)	
Yes	157 (26.4%)	131 (26.5%)	26 (26.0%)	
Ulinastatin (*n*)				0.226
No	584 (98.3%)	484 (98.0%)	100 (100%)	
Yes	10 (1.68%)	10 (2.02%)	0 (0.00%)	
Atomization (*n*)				0.328
No	554 (93.3%)	458 (92.7%)	96 (96.0%)	
Yes	40 (6.73%)	36 (7.29%)	4 (4.00%)	
ePTFE Endograft (suture technology) (*n*)				0.405
No	450 (75.8%)	378 (76.5%)	72 (72.0%)	
Yes	144 (24.2%)	116 (23.5%)	28 (28.0%)	
ePTFE Endograft (no-suture technology) (*n*)				0.678
No	512 (86.2%)	424 (85.8%)	88 (88.0%)	
Yes	82 (13.8%)	70 (14.2%)	12 (12.0%)	
Polyester Endograft (knitted process)(*n*)				**0.001**
No	558 (93.9%)	472 (95.5%)	86 (86.0%)	
Yes	36 (6.06%)	22 (4.45%)	14 (14.0%)	
Polyester Endograft (woven process) (*n*)				**0.020**
No	515 (86.7%)	436 (88.3%)	79 (79.0%)	
Yes	79 (13.3%)	58 (11.7%)	21 (21.0%)	
Atropine (*n*)				0.559
No	196 (33.0%)	160 (32.4%)	36 (36.0%)	
Yes	398 (67.0%)	334 (67.6%)	64 (64.0%)	
Flurbiprofen axetil (*n*)				0.586
No	522 (87.9%)	432 (87.4%)	90 (90.0%)	
Yes	72 (12.1%)	62 (12.6%)	10 (10.0%)	
Parecoxib (*n*)				1.000
No	543 (91.4%)	452 (91.5%)	91 (91.0%)	
Yes	51 (8.59%)	42 (8.50%)	9 (9.00%)	
Midazolam (*n*)				0.486
No	49 (8.25%)	43 (8.70%)	6 (6.00%)	
Yes	545 (91.8%)	451 (91.3%)	94 (94.0%)	
Butorphanol (*n*)				0.414
No	543 (91.4%)	449 (90.9%)	94 (94.0%)	
Yes	51 (8.59%)	45 (9.11%)	6 (6.00%)	
Dezocine (*n*)				0.987
No	252 (42.4%)	209 (42.3%)	43 (43.0%)	
Yes	342 (57.6%)	285 (57.7%)	57 (57.0%)	
Sevoflurane (*n*)				0.610
No	46 (7.74%)	40 (8.10%)	6 (6.00%)	
Yes	548 (92.3%)	454 (91.9%)	94 (94.0%)	
Deep vein puncture (*n*)				0.449
No	50 (8.42%)	44 (8.91%)	6 (6.00%)	
Yes	544 (91.6%)	450 (91.1%)	94 (94.0%)	
Radial artery puncture (*n*)				0.923
No	64 (10.8%)	54 (10.9%)	10 (10.0%)	
Yes	530 (89.2%)	440 (89.1%)	90 (90.0%)	
Vasopressor (*n*)				0.394
No	110 (18.5%)	95 (19.2%)	15 (15.0%)	
Yes	484 (81.5%)	399 (80.8%)	85 (85.0%)	
Etomidate (*n*)				**0.030**
No	63 (10.6%)	59 (11.9%)	4 (4.00%)	
Yes	531 (89.4%)	435 (88.1%)	96 (96.0%)	
Muscle relaxant (*n*)				**0.047**
Cisatracurium	544 (92.8%)	446 (91.8%)	98 (98.0%)	
Rocuronium	42 (7.17%)	40 (8.23%)	2 (2.00%)	
Ephedrine (*n*)				0.418
No	284 (47.8%)	232 (47.0%)	52 (52.0%)	
Yes	310 (52.2%)	262 (53.0%)	48 (48.0%)	
Infusion volume (ml)	1450 [1000; 2000]	1500 [1000; 2000]	1000 [1000; 2000]	0.774
Intraoperative crystal volume (ml)	500 [500; 1500]	500 [500; 1500]	1000 [500; 1500]	0.754
Intraoperative colloid volume (ml)	500 [500; 1000]	500 [500; 1000]	500 [500; 1000]	0.577
Blood loss (ml)	20.0 [20.0; 50.0]	20.0 [20.0; 50.0]	30.0 [20.0; 50.0]	0.100
Hourly urine output (ml)	40[20; 70]	40 [20; 70]	40 [20; 70]	0.592
Anesthesia duration (min)	185 [140; 235]	180 [140; 232]	205 [159; 250]	**0.020**
Surgical duration (min)	135 [100; 190]	134 [100; 180]	160 [119; 211]	**0.007**

Data were expressed as median (IQR) and frequency (proportion). Bold data indicate significance at *p* < 0.05

### Statistical analysis

Continuous variables that conform to normal distribution are described by the mean (standard deviation), while non-normal distribution uses the median (interquartile range [IQR]), and categorical data are described as counts (percentages). Continuous variables are filled with means, while categorical variables are filled with modes. All the participants were randomly divided into an 80% training set and a 20% validation set. LASSO regression was performed using the glmnet package for variable selection on the training set. Then eight classical ML algorithms, namely Multilayer Perceptron (MLP), LDA, Logistic Regression (LR), Adaptive Boosting (AdaBoost), Gaussian Naive Bayes (Gaussian NB), RandomForest (RF), eXtreme Gradient Boosting (XGBoost), and K Nearest Neighbors (KNN), were trained on the training set. The grid search method is used to determine the optimal superparameter of each algorithm based on the highest recall rate.

## Results

In our hospital, the study involved a cohort of 618 EVAR patients from January 2018 to November 2022. Of these, 24 patients were excluded from the analysis: six were excluded due to postoperative pneumonia, six were excluded due to preoperative fever or increased white blood cell count, and 12 were excluded due to significant preoperative data loss, which was identified through medical record review, laboratory testing, and imaging examination. Ultimately, 594 patients were considered for statistical analysis as the training set, revealing a PIS incidence rate of 16.8%. The flowchart is shown in Fig. 1.

### Preoperative and intraoperative characteristics of two groups

The preoperative characteristics of patients with and without PIS after EVAR are shown in Table 1.

Compared with patients in the no PIS group, the PIS group is younger in age, with higher platelet counts and absolute neutrophil count. However, there were no significant differences in gender, BMI, ASA classification, NYHA classification, hypertension, diabetes, smoking history, and other test results. The intraoperative characteristics of the two groups are shown in Table 2.

Compared with patients in the no PIS group, patients in the PIS group had more stents from polyester, received more etomidate and more muscle relaxants cisatracurium instead of rocuronium, experienced longer anesthesia and surgical times. However, there were no significant differences in the use of ePTFE, laryngeal mask or tracheal intubation general anesthesia, glucocorticoids, other anesthetic drugs, flurbiprofen axetil, parecoxib, and vasoactive drugs, as well as bleeding, infusion volume, and urine volume.

### Applying lasso regression to select features

**Fig. 2:**
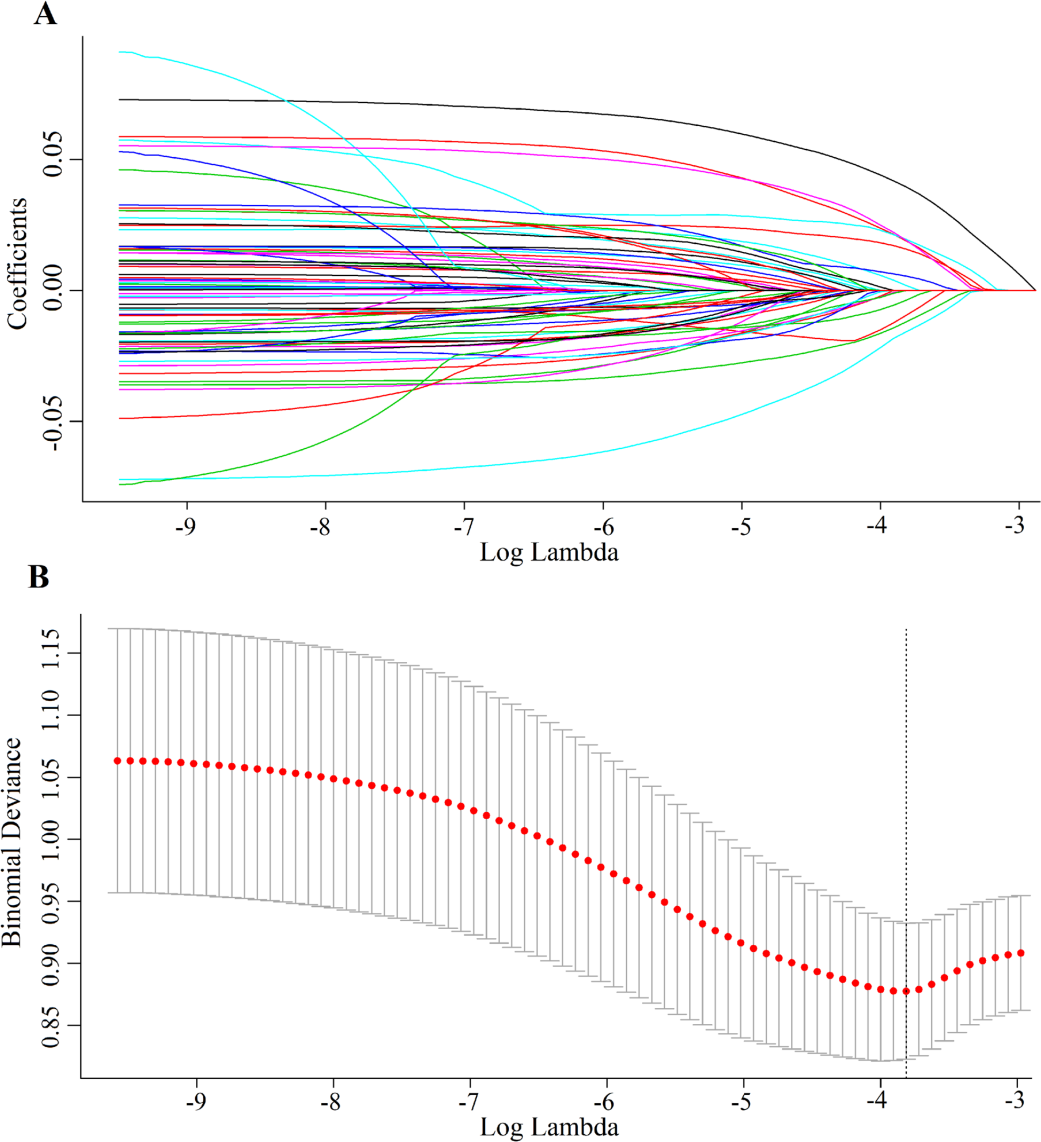
Using the least absolute shrinkage and selection operator (LASSO) regression model, 11 features were selected. **A** The coefficients of variables in the prediction model vary with the regularization parameter (log lambda), reflecting the variable selection process. **B** The trend of model performance metrics as the regularization parameter changes, where the vertical axis metric is binomial deviance, which reflects the performance of the model. The smaller the deviance, the better the predictive ability of the model, thus used to determine the optimal parameter value

Out of the last 85 features, 11 were selected that may affect the occurrence of PIS, including intraoperative use of etomidate, muscle relaxants, polyester endograf (knitted process), polyester endograf (woven process), glucocorticoids, deoxypinephrine, platelet count, age, absolute neutrophil count, surgical duration, creatinine, as shown in Fig. 2.

**Fig. 3:**
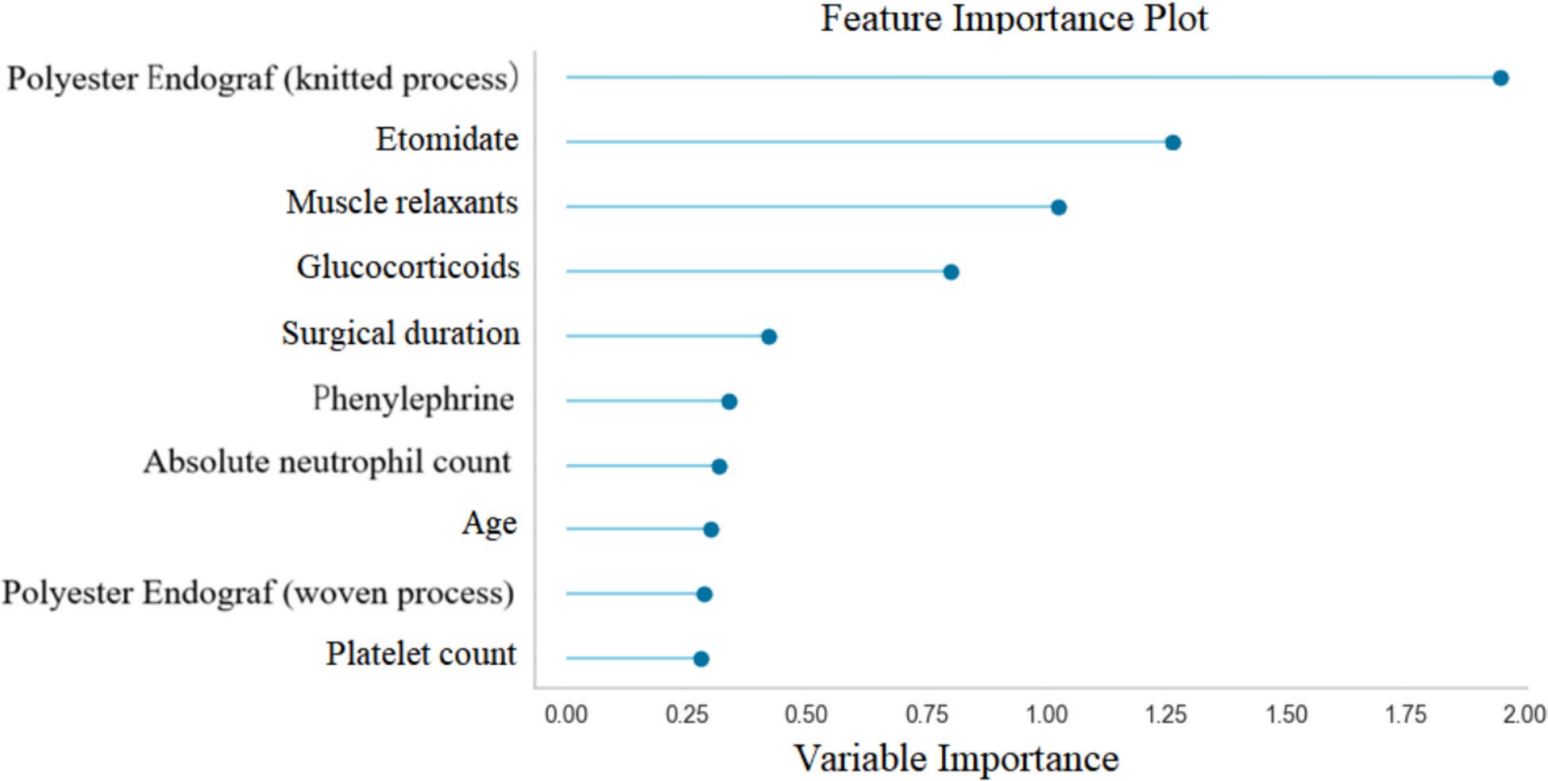
Feature importance ranking of the selected features

We created a feature importance plot and ranked the importance of features. Polyester endograf (knitted process), etomidate, and muscle relaxants ranked in the top three, as shown in Fig. 3.

### Performance evaluation of machine learning models for predicting PIS

**Fig. 4:**
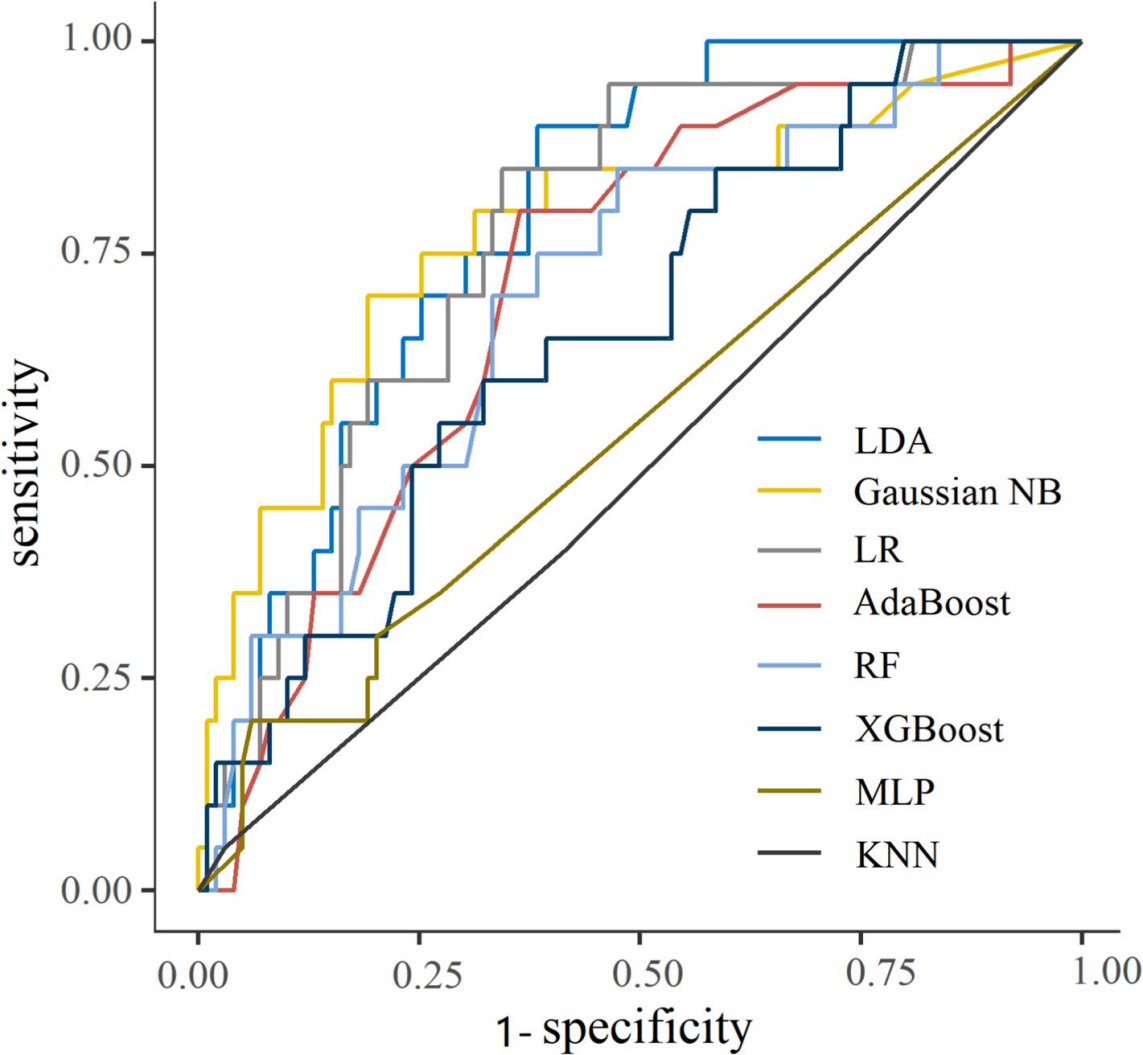
ROC curve for prediction of PIS

We evaluated the performance of eight algorithms and ultimately obtained the highest AUC (0.794, CI 0.704–0.884), second highest F1 score (0.438), correlated balanced sensitivity (0.7) and specificity (0.697), and higher accuracy (0.697) for the LDA model. Therefore, we used LDA for analysis and application, as shown in Fig. 4 and Table 3.

**Table 3:** Evaluation metrics of each model for internal validation set

Model	AUC	Cutoff^a^	Sensitivity	Specificity	Accuracy	F1 score
LDA	0.794 (0.704–0.884)	0.169	0.700	0.697	0.697	0.438
Gaussian NB	0.780 (0.656–0.904)	0.320	0.750	0.747	0.748	0.500
LR	0.774 (0.673–0.875)	0.166	0.700	0.697	0.697	0.438
AdaBoost	0.714 (0.600–0.827)	0.499	0.600	0.677	0.664	0.375
RF	0.705 (0.583–0.827)	0.170	0.650	0.667	0.664	0.394
XGBoost	0.663 (0.537–0.788)	0.049	0.600	0.606	0.605	0.338
MLP	0.546 (0.424–0.667)	0.006	0.350	0.727	0.664	0.259
KNN	0.497 (0.373–0.621)	0.333	0.400	0.586	0.555	0.232

^a^We chose a balanced cutoff between sensitivity and specificity

### Clinical utility assessment

In the internal validation set, calibration curves and precision-recall (PR) curves for the eight machine learning models are shown in Supplementary Figs. 1 and 2, respectively. Decision curve analysis across both internal and temporal validation sets demonstrated the superior clinical utility of the LDA model (Supplementary Fig. 3). Throughout the clinically relevant threshold range (10–35%), the model provided a higher net benefit than the strategies of treating all or no patients in both datasets. At the 20% decision threshold, the model yielded a net benefit of 6.7 and 5.9 per 100 patients in the internal and temporal validation sets, respectively. In contrast, the “treat-all” strategy resulted in net harm (− 4.0 per 100 patients), while the “treat-none” strategy provided no net benefit (0 per 100 patients). The consistent positive net benefit across both validation sets supports the potential of the model to guide targeted clinical interventions and optimize resource use.

## Discussion

In this study, we evaluated the ability of eight ML algorithms to predict PIS and obtained the following practical findings: (1) The incidence of postoperative PIS in EVAR was 16.8%, within the reported range of 14% and 60%, similar to the 15.8% reported by Riccardo [19]. (2) A total of 11 factors were significantly correlated with PIS, including etomidate, muscle relaxants, polyester endograf (knitted process), polyester endograf (woven process), glucocorticoids, phenylephrine, platelet count, age, absolute neutrophil count, surgical duration, and creatinine, with preoperative and intraoperative characteristics. In terms of feature importance, the top three are polyester endograf (knitted process), etomidate, and muscle relaxants. (3) Among the eight developed ML models, the LDA model performed the best in PIS prediction, with an AUC of 0.794, sensitivity of 0.7, specificity of 0.697, and accuracy of 0.697.

At present, the treatment of PIS mainly focuses on controlling the inflammatory response, but there is little literature on treatment, and no treatment algorithm has been established. The treatment effect and prognosis are still controversial. Gabriel et al. recommend actively using anti-inflammatory drugs when patients exhibit widespread clinical signs of inflammation in emergencies [20], while Morikage et al. prefer conservative methods [21]. There is still debate on whether symptomatic treatment for inflammation affects prognosis. Arnautoglou et al. suggest that research on PIS should focus on better understanding potential pathophysiology, predictive factors, and risk factors [3]. Therefore, this study collected 85 preoperative and intraoperative factors with the aim of identifying relevant risk factors, developing models to predict PIS, and achieving early clinical intervention to reduce the occurrence of PIS.

There are some studies on the analysis of PIS risk factors, but there is no application of ML methods yet. Literature reports suggest that the risk factors for PIS may be related to graft materials, mural thrombi, and contrast agents [22, 23]. There are also studies indicating that the risk factors for PIS may be related to preoperative neutrophil lymphocyte ratio, platelet count, platelet lymphocyte ratio [24], red blood cell width (RDW) [25], age, and BMI [26]. However, the sample size of the study is relatively small, and intraoperative influencing factors have not been included. This study applies ML methods to incorporate preoperative and intraoperative factors, analyze PIS risk factors, and establish predictive models, which are beneficial for anesthesiologists to conduct preoperative PIS risk assessment and intraoperative management to reduce the occurrence of postoperative PIS.

We applied the LASSO regression method to select 11 clinically widely used and routinely recorded variables for ML modeling, including preoperative patient age, platelet count, absolute neutrophil count, and creatinine; intraoperative medications include etomidate, muscle relaxants, glucocorticoids, phenylephrine, as well as surgical duration and Polyester endograf. Previous literature reports suggest that PIS may be related to coated stent materials [27–30]. Our research results found that polyester endograf is associated with higher predicted PIS risk in EVAR, and the endograf with knitted process has the greatest effect. Paquette et al. found that 33.6% of patients in the ICU experienced adrenal cortex dysfunction after the use of etomidate, while 92% of patients with adrenal cortex dysfunction experienced SIRS [31]. In this study, it was found for the first time that etomidate is the second most important risk factor associated with higher predicted PIS, and the specific mechanism is not yet clear, which may have the same mechanism as Paquette’s study. Literature reports suggest that muscle relaxants may have receptor-mediated direct anti-inflammatory effects by blocking α_1_-mediated nicotinic acetylcholine receptors [32]. Our study found that muscle relaxants have an overall protective effect on the occurrence of higher predicted PIS, but the incidence of outcomes with the use of cisatracurium is 18%, while the incidence of outcomes with the use of rocuronium is only 4.8%. The specific mechanism of the difference needs further prospective research to verify.

In our study, the linear discriminant analysis (LDA) model performed well in predicting PIS, with the highest AUC of 0.794, F1 score of 0.438, accuracy of 69.7%, and sensitivity and specificity of 69.7% and 70.0%, respectively. The linear discriminant model is a classic supervised learning algorithm with high interpretability and comprehensibility, which can provide intuitive results. The proposed model could potentially transform post-EVAR management in several ways: First, high-risk patients (predicted probability above the clinical threshold) could receive targeted steroid prophylaxis, potentially mitigating the inflammatory cascade. Second, these patients could be monitored more closely for fever, leukocytosis, and hemodynamic changes, enabling earlier intervention. Third, low-risk patients could avoid unnecessary laboratory testing and empirical therapies, optimizing resource allocation. The decision curve analysis supports these applications by demonstrating net clinical benefit within the threshold range where such interventions would typically be considered.

Our research has several limitations that should not be ignored. First, this single-center study used only internal validation; the lack of external validation limits the model’s generalizability, and multicenter studies are planned to verify our results. Second, postoperative case data were not included in the analysis, as the study focused on the early prediction and intervention of PIS rather than on delayed postoperative events. Third, respiratory parameters from the anesthesia machine were unavailable, so they were not incorporated into the study, and their potential impact on PIS cannot be ruled out. Fourth, only data on early PIS within 72 h after EVAR were analyzed, which provides no insights into delayed postoperative inflammatory responses. Fifth, the exclusion criteria did not exclude diseases such as malignancy, hematologic diseases, systemic autoimmune disorders, and aortic rupture. Future multicenter prospective studies with external validation, comprehensive perioperative data collection, and stricter exclusion criteria are needed to confirm our findings.

## Conclusions

This research has developed an ML prediction model for the occurrence of PIS based on the LDA algorithm. The selected features include preoperative and intraoperative variables, which are expected to reduce the occurrence of postoperative PIS in EVAR by controlling these variables in clinical practice.

## Supplementary Information

Supplementary Material 1.

## Data Availability

The data supporting the findings of this study is available from the corresponding author upon request.
