# Supplementary material for: Developing a practical machine learning model to predict post implantation syndrome after endovascular aneurysm repair
Source: CVIR Endovasc. 2026 Mar 18;9:29. doi: 10.1186/s42155-026-00668-w (PMC13000017; doi:10.1186/s42155-026-00668-w)

**Supplementary table 1. main characteristics of temporal validation set**

| **Characteristics** | **All population (N=312)** | **no-PIS （N=261）** | **PIS （N=51）** | ***p* value** |
| --- | --- | --- | --- | --- |
| Age (y) | 71.0 [64.8;76.0] | 71.0 [64.0;76.0] | 72.0 [65.5;75.5] | 0.834 |
| Platelet (10^9^/L) | 231 [181;283] | 230 [183;276] | 237 [178;317] | 0.52 |
| Creatinine (μmol/L) | 87.0 [71.8;111] | 86.0 [71.0;107] | 100 [74.0;121] | 0.064 |
| Absolute neutrophil count(10^9^/L) | 5.12 [3.99;6.60] | 4.86 [3.88;6.11] | 6.18 [4.72;7.51] | 0.001 |
| Glucocorticoids(n) |  |  |  | 0.043 |
| No | 303 (97.1%) | 256 (98.1%) | 47 (92.2%) |  |
| Yes | 9 (2.88%) | 5 (1.92%) | 4 (7.84%) |  |
| Phenylephrine (n) |  |  |  | <0.001 |
| No | 262 (84.0%) | 236 (90.4%) | 26 (51.0%) |  |
| Yes | 50 (16.0%) | 25 (9.58%) | 25 (49.0%) |  |
| Polyester Endograft(knitted process) (n) |  |  |  | <0.001 |
| No | 252 (80.8%) | 228 (87.4%) | 24 (47.1%) |  |
| Yes | 60 (19.2%) | 33 (12.6%) | 27 (52.9%) |  |
| Polyester Endograft(woven process) (n) |  |  |  | 0.022 |
| No | 253 (81.1%) | 218 (83.5%) | 35 (68.6%) |  |
| Yes | 59 (18.9%) | 43 (16.5%) | 16 (31.4%) |  |
| Etomidate (n) |  |  |  | <0.001 |
| No | 206 (66.0%) | 198 (75.9%) | 8 (15.7%) |  |
| Yes | 106 (34.0%) | 63 (24.1%) | 43 (84.3%) |  |
| Muscle relaxant (n) |  |  |  | <0.001 |
| Cisatracurium | 211 (67.6%) | 201 (77.0%) | 10 (19.6%) |  |
| Rocuronium | 101 (32.4%) | 60 (23.0%) | 41 (80.4%) |  |
| Surgical duration(min) | 135 [105;190] | 138 [105;190] | 125 [106;173] | 0.327 |

| **Supplementary table 2. Best hyperparameter chosen by using GridSearchCV based on setting of hyperparameter range** | | |
| --- | --- | --- |
| **Model** | **Setting of hyperparameters** | **Best hyperparameter values for outcome prediction** |
| LDA | solver: [svd, lsqr, eigen] tol: [0.0001,0.0002,0.0003] | solver=svd tol=0.0001 |
| Gaussian NB | - | - |
| LR | penalty:[L1, L2] C:[0.1, 1, 10, 100, 200, 500, 1000] | penalty=L2 C=100 |
| AdaBoost | n_estimators: [10, 100, 1000, 10000] learning_rate: [0.001, 0.01, 0.1, 1] | n_estimators=10000 learning_rate=1 |
| RF | n_estimators:[10, 100, 1000, 10000] max_features:[auto, log2] max_depth:[2, 5, 10, 20, 50, 100] criterion:[gini, entropy] | n_estimators=10 max_features=auto max_depth=10 criterion=gini |
| XGBoost | max_depth:[2, 3, 10, 30, 100] n_estimators:[15, 150, 500, 1000,1500, 15000] learning_rate:[0.001, 0.01, 0.1, 0.2, 1] eval_metric:[mlogloss] | max_depth=2 n_estimators=1000 learning_rate=0.1 eval_metric=mlogloss |
| MLP | solver: [lbfgs, adam] learning_rate: [constant, invscaling, adaptive] activation: [identity, logistic, tanh, relu] | solver=lbfgs learning_rate=constant activation=tanh |
| KNN | n_neighbors:[3, 5, 10, 20] leaf_size:[2, 5, 10, 20] p:[0.5, 1, 2, 5] weights:[uniform, distance] | n_neighbors=3 leaf_size=2 p=1 weights=uniform |

(Supplementary Table 2). We compared the performance of each model using the AUC, sensitivity, specificity, accuracy and F1 value, then the optimal model was chosen for validation on the validation set. Data preprocessing and variable selection based LASSO were performed by R-4.1.0 (R Foundation for Statistical Computing, Vienna, Austria), and machine learning model build was performed by Python (version 3.7.11, Python Software Foundation). All results were considered statistically significant at p<0.05.

**Supplementary table 3. Evaluation metrics of each model for temporal validation set**

| **Model** | **AUC** | Cutoff **^*^** | **Sensitivity** | **Specificity** | **Accuracy** | **F1 score** |
| --- | --- | --- | --- | --- | --- | --- |
| Linear Discriminant Analysis (LDA) | 0.814 (0.748, 0.880) | 0.169 | 0.647 | 0.851 | 0.817 | 0.537 |
| Gaussian Naive Bayes (Gaussian NB) | 0.647 (0.554, 0.740) | 0.320 | 0.078 | 0.927 | 0.788 | 0.108 |
| Logistic Regression (LR) | 0.800 (0.732, 0.868) | 0.166 | 0.510 | 0.874 | 0.814 | 0.473 |
| Adaptive Boosting (AdaBoost) | 0.866 (0.814, 0.919) | 0.499 | 0.059 | 0.996 | 0.843 | 0.109 |
| RandomForest (RF) | 0.763 (0.698, 0.827) | 0.170 | 0.667 | 0.709 | 0.702 | 0.422 |
| eXtreme Gradient Boosting (XGBoost) | 0.765 (0.689, 0.842) | 0.049 | 0.765 | 0.586 | 0.615 | 0.394 |
| Multilayer Perceptron (MLP) | 0.491 (0.391, 0.591) | 0.006 | 0.824 | 0.111 | 0.228 | 0.258 |
| K Nearest Neighbors (KNN) | 0.754 (0.678, 0.831) | 0.333 | 0.392 | 0.904 | 0.821 | 0.417 |

***Cutoff obtained based on internal validation set**

**Supplementary figure 1. Calibration curves of 8 machine learning models in the internal validation set.** Calibration curves showing the agreement between predicted probabilities and observed outcomes for each model. The red dashed line represents perfect calibration (y = x), the blue line shows the LOESS-smoothed calibration curve, and gray points indicate individual predictions. Each subplot displays the calibration slope, C-statistic, and Brier score.


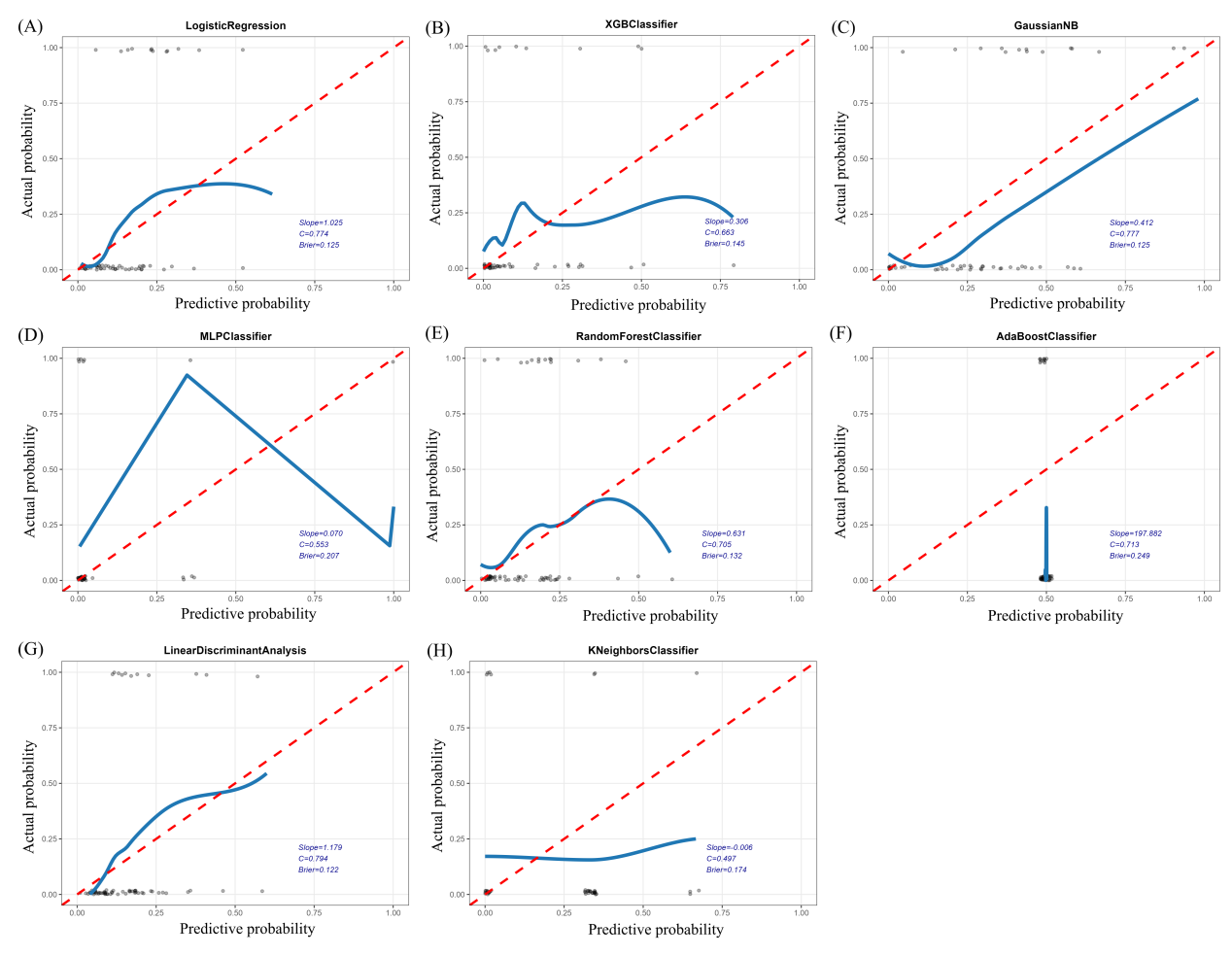


**Supplementary figure 2. Precision-recall curves of 8 machine learning models in the internal validation set.** The curves illustrate the relationship between precision (positive predictive value) and recall (sensitivity) across different classification thresholds. Each colored line represents one machine learning model, with performance ranked by the area under the precision-recall curve (AUPRC). The gray dashed line indicates the performance of a random classifier (AUPRC = 0.168).


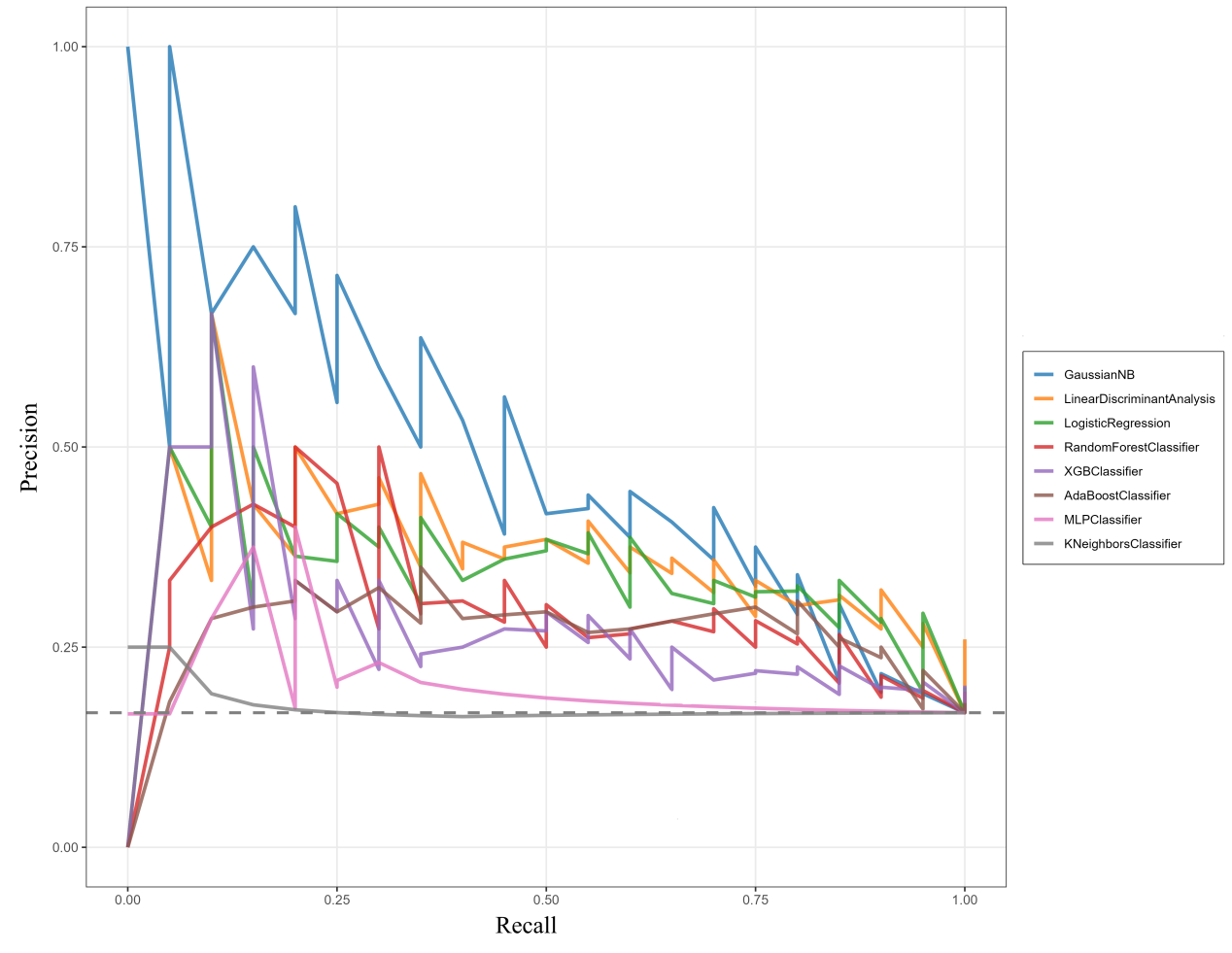


**Supplementary figure 3. Decision curve analysis of the LDA model for predicting PIS.**

Internal validation set. (B) Temporal validation set. The LDA model (solid line) demonstrates superior net benefit compared to the "treat-all" (dashed line) and "treat-none" (dotted line) strategies across a threshold probability range of 10% to 35%.


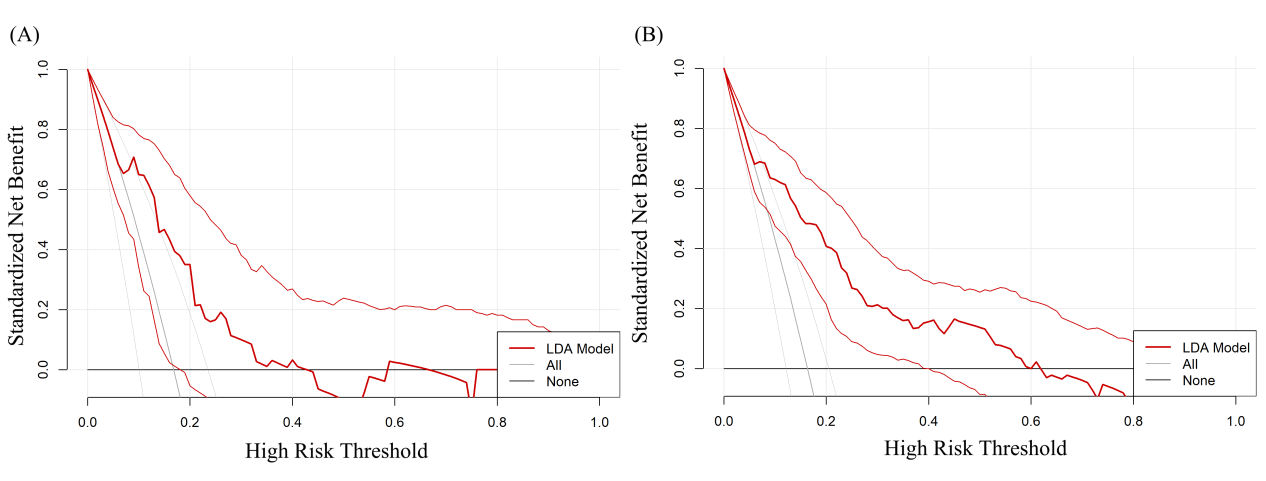

Supplement: Supplementary file 1 — Supplementary Material 1. [file 42155_2026_668_MOESM1_ESM.docx]
